# Natural products regulate mitochondrial function in cognitive dysfunction—A scoping review

**DOI:** 10.3389/fphar.2023.1091879

**Published:** 2023-03-07

**Authors:** Jinmei Tuo, Yan Peng, Yushuang Linghu, Ming Tao, Shiming Huang, Zucai Xu

**Affiliations:** ^1^ Department of Neurology, Affiliated Hospital of Zunyi Medical University, Zunyi, China; ^2^ Department of Nursing, Affiliated Hospital of Zunyi Medical University, Zunyi, China; ^3^ The Collaborative Innovation Center of Tissue Damage Repair and Regeneration Medicine of Zunyi Medical University, Zunyi, China

**Keywords:** natural products, mitochondrial function, cognitive dysfunction, scoping review, neurodegenerative diseases

## Abstract

Medicines from natural products can not only treat neurodegenerative diseases but also improve the cognitive dysfunction caused by treatments with western medicines. This study reviews the literature related to the regulation of mitochondrial participation in cognitive function by natural products. In this study, we focused on English articles in PubMed, Web of Science, and Google Scholar, from 15 October 2017, to 15 October 2022. Fourteen studies that followed the inclusion criteria were integrated, analyzed, and summarized. Several studies have shown that natural products can improve or reduce cognitive dysfunction by ameliorating mitochondrial dysfunction. These results suggest that natural products may serve as new therapeutic targets for neurodegenerative diseases.

## 1 Introduction

Natural products contain a wide range of biologically active metabolites (Park et al., 2021). These compounds usually originate from complex biosynthetic systems and exhibit various morphological characteristics (Nair and Jez, 2020). They can be used in pharmaceutical research and development and drug design, as well as in dietary supplements, natural foods without artificial ingredients, and cosmetics (Newman and Cragg, 2012; Nair and Jez, 2020; Park et al., 2021). In this study, we focused on botanical drugs derived from natural products. Natural products are characterized by their chemical diversity and complexity. Many metabolites contain a large number of stereo-specific carbon centers. Thus, an increasing number of scientists are interested in the stereo-complexity of these molecules (Katz and Baltz, 2016). With the continuous development of natural product research, which began with the discovery and medical application of antibiotics in the 1950s, the average lifetime of the population increased significantly over the following 10–15 years (Bérdy, 2012). Katz and Baltz divided the history of natural product discovery into three overlapping periods, from the 1940s–1970s, 1970s–2000s, and beyond 2000 according to their impact on the discovery of new natural products. The first 30 years, the phenotypic screening stage, mainly focused on the discovery of antibacterial and antifungal metabolites; the second 30 years encompassed a huge expansion of screening methods and strategies; the third 30 years incorporated genomics-based approaches (Katz and Baltz, 2016). With the rapid development of science and technology, machine learning models have been used to predict the biochemical and physiological effects of natural products (Jeon et al., 2021). Hence, machine learning models are expected to have a significant impact on the future development and research of the molecular structures of natural products.

Mitochondria are multifunctional organelles (Nunnari and Suomalainen, 2012; Chandel, 2014) that have multiple functions, from bioenergy to cell signaling, and are signaling centers that induce transcription, proteomic, and posttranslational regulation mechanisms (Khacho and Slack, 2017). Mitochondrial function and mitochondrial dynamics play important roles in the process of neural development. Mitochondria play a central role in determining the fate of neural stem cells (NSCs) (Khacho et al., 2019). In the central nervous system, mitochondrial diseases are characterized by cognitive dysfunction, which leads to behavioral abnormalities (Fattal et al., 2006). Metabolic disorders caused by mutations in mitochondrial or nuclear DNA often affect many organs and systems. Among them, nervous system symptoms are the most common and most serious (McFarland et al., 2010), and they manifest as epilepsy, cognitive dysfunction, progressive dementia, and lactic acidosis (Kartsounis et al., 1992; Turconi et al., 1999). Mitochondria maintain their total number and morphology mainly through the regulation of the counterbalance between fission and fusion, namely mitochondrial dynamics. Therefore, changes in mitochondrial morphology and function are indispensable in the process of neurogenesis.

The mitochondria facilitate adenosine triphosphate (ATP) production, calcium regulation, and redox maintenance, hence their dysfunction can lead to various neurodegenerative diseases, including Alzheimer’s disease (AD) (Xia et al., 2018). Mitochondrial and synaptic dysfunction has been proven to be an early symptom of AD (Pérez et al., 2018). To date, there is no standard treatment for mitochondrial disorders. With the development of medical treatments, multiple pharmacological properties of botanical drugs have been applied to study neurodegenerative diseases, such as salidroside and astragalus polysaccharides, which have a protective effect on cognitive-related changes (Zhang et al., 2019; Xie et al., 2020). Asiatic acid can prevent and alleviate seizures (Lu et al., 2021).

Currently, an increasing number of natural product extracts involved in mitochondrial regulation have attracted attention, including their effects on mitochondrial dynamics and autophagy. Therefore, this study reviews the literature on natural products’ regulation of mitochondrial function in cognitive dysfunction diseases to clarify the possible mechanism underlying mitochondrial function as influenced by natural products, identify new therapeutic targets for the treatment of neurodegenerative diseases, and provide an insight into the development of natural products for the treatment of cognitive impairment.

## 2 Methods

### 2.1 Search strategy

**FIGURE 1:**
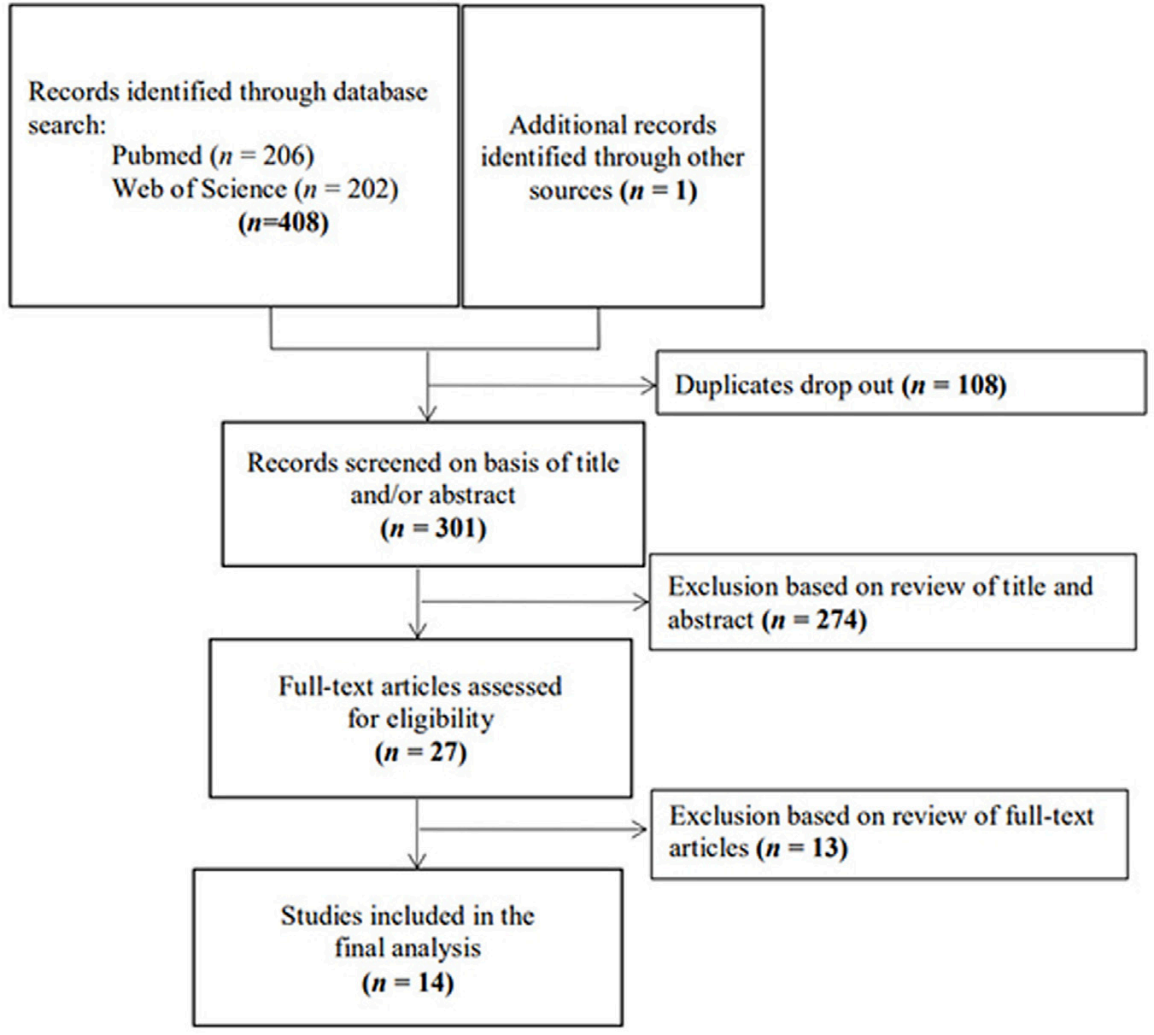
Flow chart of article identification.

In this study, we used various keywords to search a range of English databases. We used “natural products,” “mitochondrial,” “mitochondrial function,” “nerve cell,” “neurocyte,” “cognitive dysfunction,” “Alzheimer,” and “dementia” as keywords for search and retrieval. We focused on English articles in PubMed, Web of Science, and Google Scholar from 15 October 2017, to 15 October 2022. The details of this information are shown in Figure 1. Studies that followed the inclusion criteria of the present study were integrated, analyzed, and summarized.

### 2.2 Data extraction

In the present study, the articles were independently screened and identified by two authors (JT and YL). At the same time, according to the data collection sheet (Supplementary File) designed by the two authors, the first author independently extracted the data that included the author, year, country, type of study, name of natural products, mechanism of regulating mitochondrial function in cognitive dysfunction, and main results (Colquhoun et al., 2014). All authors checked the accuracy of the extracted data.

### 2.3 Inclusion and exclusion criteria

The inclusion criteria of the literature were determined before searching by JT and ZX to include only studies related to our research questions. The inclusion criteria were as follows: 1) English language studies, 2) articles published in scientific journals, 3) articles containing natural products, 4) studies on the impact of mitochondria on cognitive function, and 5) studies discussing the possible mechanism of correlation with neurological disorders. Articles that did not meet the inclusion criteria were excluded.

### 2.4 Botanical drugs used in the present study

*Erigeron* (Dengzhanxixin) [Asteraceae; Erigeron breviscapus (Vaniot) Hand.-Mazz.] injections (DZXI) (specifications: 5.32 mg scutellarin, 2.26 mg 3,4-O-dicaffeoylquinic acid, 1.10 mg 3,5-O-dicaffeoylquinic acid, 1.79 mg erigoster B, 2.70 mg 4,5-O-dicaffeoylquinic acid, and 11.26 mg erigeroster, 10 mL/ampoule, Lot No. 20180137) were provided by Yunnan Biovalley Pharmaceutical Co., Ltd. (Kunming, China), erigeroster (Lot No, 20190701, purity 93.3%) were provided by Yunnan Biovalley Pharmaceutical Co., Ltd. (Kunming, China) and Chengdu Pusi Biotechnology Co., Ltd. (Chengdu, China) (An et al., 2021).

Dried leaves of *Centella* [Apiaceae; *Centella asiatica* (L.) Urban] (Oregon’s Wild Harvest, GOT-03193c-OHQ01) was used. CAW (*Centella asiatica*) was prepared by refluxing CAW (160g) with water (2,000 mL) for 2 h, filtering the solution, and freeze drying to yield a powder (∼16–21 g) (Gray et al., 2018).

Asiatic acid is a triterpene derived from the medicinal botanical drug *Centella*[Apiaceae; *Centella asiatica* (L.) Urban]. It included Asiatic acid (purity >99%, Aktin, A98688, Chengdu, China) and kainic acid (Sigma-Aldrich, K0250, St. Louis, MO, USA) (Lu et al., 2021).

*Actinidia* [Actinidiaceae; Actinidia arguta (Siebold & Zucc.) Planch. ex Miq.] was obtained a hardy kiwi from the National Institute of Forest Science (Suwon, Korea) in September 2013, and it was extracted in 40% ethanol at 40°C for 2 h. It was obtained by filtration, evaporation, freezing, and drying (Ha et al., 2020).

Tetramethylpyrazine (TMP) was extracted from the rhizome of *Ligusticum chuanxiong* [Apiaceae; Conioselinum anthriscoides “Chuanxiong”] (Huang et al., 2021). The extraction method was not clearly explained.

*Schisandra* [Schisandraceae; Schisandra chinensis (Turcz.) Baill.] was purchased in Seoul, Korea in September 2017. It was ground and powdered, extracted with 70% EtOH two times at room temperature, then extracted by Diaion HP-20 chromatography and preparative high-performance liquid chromatography (HPLC) (column: YMC-Pack ODS-A, 5 μm, 250 × 20 mm I.D., Japan, 8 mL/min, 10%–35% MeCN, 40 min) (Jang et al., 2020).

(−) – Epicatechin ≥90% (HPLC, EC, Sigma, USA, E1753) is a metabolite (Ling et al., 2022).

Ginkgolide is a natural product from *Ginkgo* [Ginkgoaceae; Ginkgo biloba L.] leaves. Ginkgolide K (GK) is a metabolite from ginkgolide and diterpene lactone (Liu et al., 2022a).

Capsaicin is metabolite from *Capsicum* [Solanaceae; Capsicum annuum L.] (Ouyang et al., 2022). The extraction method was not clearly explained (Ouyang et al., 2022).

Trans-cinnamaldehyde (CIN) is a natural product from *Cinnamomum* [Lauraceae; Cinnamomum verum J.Presl]. Ellagic acid (ELA) is a metabolite. The extraction method was not clearly explained (Pan et al., 2020).

Red ginseng (RG) is a therapeutic material that *Panax ginseng* [Araliaceae; Panax ginseng C.A.Mey.] (PG) Meyer obtained by steaming and drying. The extraction method was not clearly explained (Shin et al., 2019).

ShenmaYizhi decoction (SMYZD) (composition: *Panax ginseng* [Araliaceae; Panax ginseng C.A.Mey.], *Gastrodia elata* [Orchidaceae; Gastrodia elata Blume], *Asplenium* [Asteraceae, Asplenium thunbergii Kunze], and *Ligusticum chuanxiong* [Apiaceae; Conioselinum anthriscoides ‘Chuanxiong'] (Chuanxiong) were prepared in a ratio of 3:3:3:2 at Xiyuan Hospital of China, Academy of Chinese Medical Sciences, Beijing Hospital preparation (Beijing Medicine preparation: Z20200005000) (Sun et al., 2021).

Rhein is a metabolite from *Rheum* [Polygonaceae; Rheum officinale Baill.] that was purchased from Shanghai Aladdin Biochemical Technology Co., Ltd. (Shanghai, China) (Yin et al., 2021; Yin et al., 2022).

### 2.5 Analysis

We searched the literature library, and 14 articles were finally included in our analysis according to our inclusion criteria. We summarized and sorted the data of the 14 articles and analyzed and discussed what is known about the effect of natural products on mitochondria in cognitive impairment.

## 3 Results

In the present study, 408 articles were initially retrieved from the database. An additional article related to the purpose of the present study was found through a manual search. A total of 108 repetitive articles were excluded (96 duplicate articles automatically removed by EndNote software, and 12 articles were excluded manually), 274 articles were excluded through article titles and abstract screening, and 13 articles were dropped after full-text screening as they did not meet the purpose of this study. Fourteen articles were included in the final analysis (Figure 1).

### 3.1 Characteristics of the included studies

The characteristics of the included studies are summarized in the Supplementary File. Of the 14 articles, one article was from the USA (Gray et al., 2018), nine from China (Pan et al., 2020; An et al., 2021; Huang et al., 2021; Sun et al., 2021; Yin et al., 2021; Liu et al., 2022b; Ling et al., 2022; Ouyang et al., 2022; Yin et al., 2022), two from Korea (Shin et al., 2019; Jang et al., 2020), and one from Taiwan (Lu et al., 2021).

All articles used animal experiments except one, which was a mixed clinical trial (An et al., 2021). Among the natural products investigated were DZXI (An et al., 2021), CAW (Gray et al., 2018; Lu et al., 2021), Chloroform fraction *Actinidia* arguta (CFAA) (Ha et al., 2020), TMP from *Ligusticum chuanxiong* (Huang et al., 2021), *Schisandra* chinensis extract (SCE), ascorbic acid (AA) (Jang et al., 2020), (−)-Epicatechin (Epi) from *Green tea* (Ling et al., 2022), GK from *Ginkgo* biloba (Liu et al., 2022a), Capsaicin from *Capsicum* (Ouyang et al., 2022), CIN and ELA from *Cinnamomum, Metabolite* (Pan et al., 2020), Red ginseng extract (RGE) (Shin et al., 2019), SMYZD from *Panax ginseng, Gastrodia elata* (Sun et al., 2021), *Rheum* (Yin et al., 2022), and Rhein, emodin, aloe-emodin, chrysophanol, and physcion from *Rheum* (Yin et al., 2021).

### 3.2 Association between natural products acting on mitochondria and cognitive function

The summary results in the Supplementary File show that there is a close relationship between mitochondria and nervous system diseases, and that the natural products of Chinese botanical drugs have protective effects on mitochondria.

Among them, DZXI (Erigeron) treatment can protect mitochondrial functions and increase the resistance of neurons to neurodegeneration (An et al., 2021). Asiatic acid (*Centella*) can not only prevent damage to the hippocampal synaptic mitochondrial structure in epileptic rats but can also affect mitochondrial proteins related to energy generation (Gray et al., 2018; Lu et al., 2021). CFAA (*Actinidia*) can effectively protect against high glucose (HG)-induced neurotoxicity, thereby improving cognitive function (Ha et al., 2020). TMP (*Ligusticum chuanxiong*) treatment reduces amyloid β (Aβ) accumulation in an AD model, promotes the recovery of mitochondrial function in mice, and improves synaptic dysfunction (Huang et al., 2021). The combination of SCE and AA (*Schisandra*) resulted in higher GluR1 levels in the hippocampus of eight-week-old male C57BL/6 mice than in that of mice injected with SCE or AA alone and enhanced mitochondrial respiration of hippocampal neurons (Jang et al., 2020). Epi upregulated the expression of phosphorylation of adenosine 5′-monophosphate-activated protein kinase (pAMPK) and lost its protective effect in cells blocked by adenosine 5′-monophosphate-activated protein kinase (AMPK). Epi had a protective effect in lipopolysaccharide-induced cells and mouse models (Ling et al., 2022). GK (*Ginkgo*) promotes neuronal cell survival and prevents apoptosis (Liu et al., 2022b). Capsaicin (*Capsicum*) exerts a neuroprotective effect on Chronic cerebral hypoperfusion-induced cognitive impairment (Ouyang et al., 2022). The combination of ELA and CIN (*Cinnamomum, Metabolite*) had a strong and consistent impact on cognitive function in rats (Pan et al., 2020). RGE (*Panax ginseng*) can restore the respiratory capacity of damaged mitochondria and prevent mitochondrial dysfunction (Shin et al., 2019). SMYZD (*Panax ginseng, Gastrodia elata, Asplenium, Ligusticum chuanxiong*) can effectively improve the structure and energy metabolism of mitochondria, thereby improving cerebral perfusion insufficiency (Sun et al., 2021). Rhein (*Rheum*) improved mitochondrial biogenesis, exhibited good antioxidant activity, and significantly increased superoxide dismutase activity (Yin et al., 2021, 2022).

### 3.3 Mechanism by which natural products regulate mitochondrial function in cognitive dysfunction

DZXI (*Erigeron*) treatment improved the level of cytochrome C oxidase subunit 2 by regulating the mitochondrial electron transport chain, thus protecting the stability of mitochondria in neural cells (An et al., 2021). CAW (*Centella*) increased synaptic vesicle exocytosis by kainic acid-induced presynaptic mitochondria and increased ATP production of hippocampal neurons (Gray et al., 2018). It can inhibit glutamate release, increase AKT activation, inhibit protease activation, protect synaptic and mitochondrial functions, and prevent cognitive deficits in kainic acid-induced epilepsy (Lu et al., 2021). CFAA (*Actinidia*) can effectively improve high-fat diet-induced mitochondrial dysfunction, thereby improving cognitive dysfunction (Ha et al., 2020). TMP (*Ligusticum chuanxiong*) treatment can significantly change mitochondrial protein levels, increase ATP levels, and achieve neuroprotection by inhibiting the activity of BACE1 (Huang et al., 2021). An SCE-AA (*Schisandra*) mixture can improve cognitive ability by enhancing mitochondrial respiration, and its short-term treatment can reduce the expression of PSD95 (Jang et al., 2020). Epi protects mitochondria by activating AMPK signaling in sepsis-associated encephalopathy (Ling et al., 2022). Treatment with GK (*Ginkgo*) can reduce the mitochondrial Ca^2+^ uniporter (MCU) induced by Aβ *in vitro*, reduce the level of Ca^2+^ in the mitochondria, inhibit cell apoptosis, and improve cognitive dysfunction (Liu et al., 2022a). Capsaicin (*Capsicum*) has a neuroprotective effect on cognitive dysfunction caused by chronic cerebral hypoperfusion (Ouyang et al., 2022). RGE (*Panax ginseng*) enhances mitochondrial respiratory function, mediates the recovery of mitochondrial function, and prevents Aβ deposition and related pathologies (Shin et al., 2019). SMYZD (*Panax ginseng, Gastrodia elata, Asplenium, Ligusticum chuanxiong*) plays a therapeutic role by activating AMPK/PPARα to restore and improve the structure and function of mitochondria by inhibiting the oxidation reaction (Sun et al., 2021). Rhein (*Rheum*) plays an active role in regulating enzymes and antioxidant enzymes in the respiratory chain complex to improve mitochondrial biogenesis, reduce the release of cytochrome C, inhibit the apoptosis cascade, and protect neurons from apoptosis (Yin et al., 2021; Yin et al., 2022) (Table 1).

**TABLE 1:** Mechanisms underlying natural product regulation of mitochondrial function in cognitive dysfunction.

Author	Country of origin	Type of study	Name of nature products	Mechanism of regulating mitochondrial function in cognitive dysfunction
An et al. (2021)	China	Clinical Trial and Animal Experiment	Erigeron (Dengzhanxixin)	DZXI treatment improved the level of cytochrome C oxidase subunit 2 by regulating the mitochondrial electron transport chain, thus protecting the stability of mitochondria in neural cells
Gray et al. (2018)	USA	Animal Experiment	Centella	Centella asiatica increased synaptic vesicle exocytosis by kainic acid-induced presynaptic mitochondria and increased ATP production of hippocampal neurons
Lu et al. (2021)	Taiwan	Animal Experiment	Centella	Centella asiatica inhibited glutamate release, increased AKT activation, inhibited protease activation, protected synaptic and mitochondrial functions, and prevented cognitive deficits in kainic acid-induced epilepsy
Ha et al. (2020)	Korea	Animal Experiment	Actinidia	Chloroform fraction Actinidia argut effectively improved high-fat diet-induced mitochondrial dysfunction, thereby improving cognitive dysfunction
Huang et al. (2021)	China	Animal Experiment	Ligusticum chuanxiong	Tetramethylpyrazine treatment significantly changed mitochondrial protein levels, increased ATP levels, and achieved neuroprotection by inhibiting the activity of BACE1
Jang et al. (2020)	Korea	Animal Experiment	Schisandra	An SCE-AA mixture improved cognitive ability by enhancing mitochondrial respiration, and its short-term treatment can reduce the expression of PSD95
Ling et al. (2022)	China	Animal Experiment	Metabolite	Epi protected mitochondria by activating AMPK signaling in sepsis-associated encephalopathy
Liu et al. (2022b)	China	Animal Experiment	Ginkgo	Treatment with GK reduced the MCU induced by Aβ *in vitro*, reduced the level of Ca^2+^ in the mitochondria, inhibited cell apoptosis, and improved cognitive dysfunction
Ouyang et al. (2022)	China	Animal Experiment	Capsicum	Capsaicin exerts a neuroprotective effect on Chronic cerebral hypoperfusion-induced cognitive impairment
Pan et al. (2020)	China	Animal Experiment	Cinnamomum, Metabolite	Combination therapy improved mitochondrial function by reducing the mitochondrial ROS production and mitochondrial membrane depolarization, increasing cellular ATP production, declining inflammatory cytokines, and lessening cell apoptosis in rats
Shin et al. (2019)	Korea	Animal Experiment	Panax ginseng	RGE enhanced mitochondrial respiratory function, mediated the recovery of mitochondrial function, and prevents Aβ deposition and related pathologies
Sun et al. (2021)	China	Animal Experiment	Panax ginseng, Gastrodia elata, Asplenium, Ligusticum chuanxiong	SMYZD played a therapeutic role by activating AMPK/PPARα to restore and improve the structure and function of mitochondria by inhibiting the oxidation reaction
Yin et al. (2022)	China	Animal Experiment	Rheum	The rhein improved mitochondrial biogenesis, recover mitochondrial dynamics, repair damaged mitochondria, and inhibit the production of ROS from ETC.
Yin et al. (2021)	China	Animal Experiment	Rheum	Rhein played an active role in regulating enzymes and antioxidant enzymes in the respiratory chain complex to improve mitochondrial biogenesis, reduce the release of cytochrome C, inhibit the apoptosis cascade, and protect neurons from apoptosis

Aβ, β-Amyloid; AKT, protein kinase B; ROS, reactive oxygen species; ATP, adenosine triphosphate; BACE, beta-secretase; AMPK, adenosine 5′-monophosphate-activated protein kinase; PSD, postsynaptic density protein; MCU, mitochondrial Ca2+ uniporter; SCE, schisandra chinensis extract; ETC, electron transport chain; AA, ascorbic acid; GK, ginkgolide K; RGE, red ginseng extract; SMYZD, shenmaYizhi decoction.

## 4 Discussion

**FIGURE 2:**
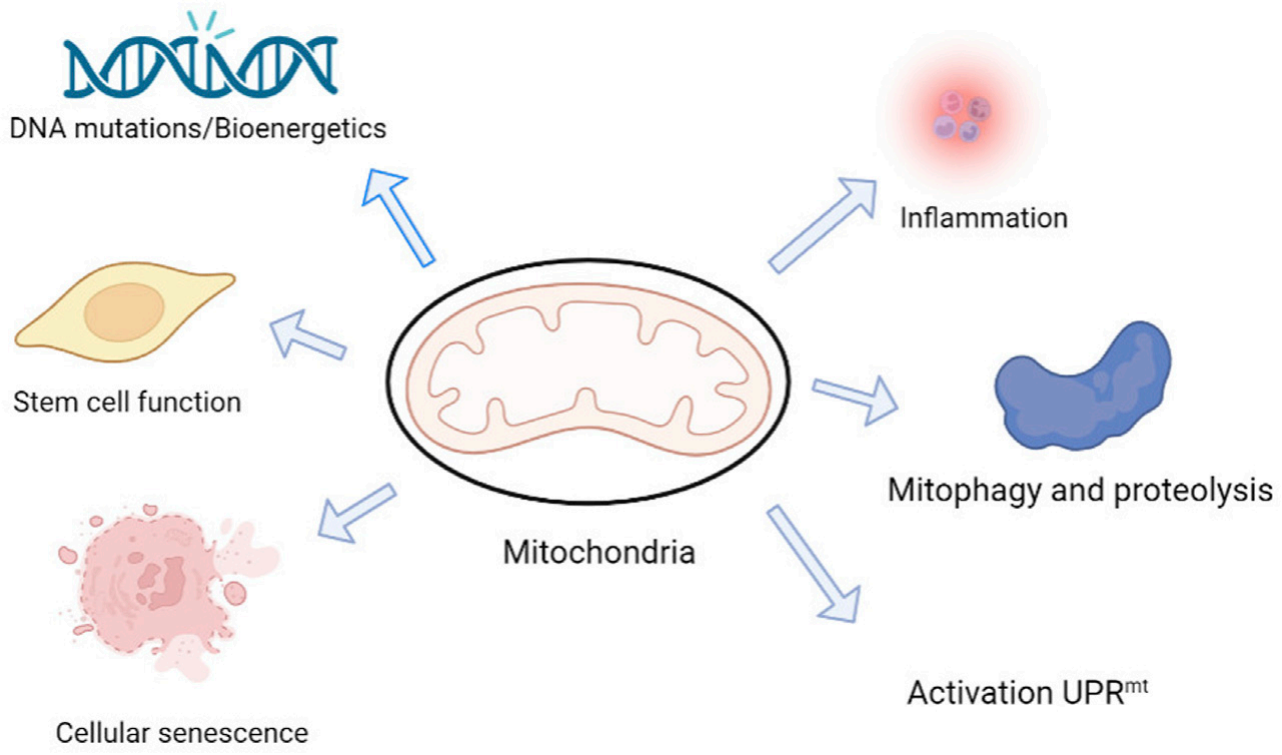
Mitochondria are possibly involved in physiological and pathological mechanisms underlying cognition changes in neurodegeneration and aging. Mitochondria are multifunctional organelles with multiple functions, from bioenergy to cell signaling, and are signaling centers that induce transcription, proteomic, and posttranslational regulation mechanisms. The mitochondria play a central role in determining the fate of neural stem cells (NSCs). Mitochondria are also involved in protein assembly and regulation and mediate cell proliferation, differentiation, and death. Mitochondria can initiate a protective program called the mitochondrial unfolded protein response (UPRmt) and use this protective mechanism to maintain normal mitochondrial function (Liu et al., 2022b).

Mitochondria are multifunctional organelles with an independent genome, and mutations to their genes and structural damage are key factors underlying cognitive impairment in neurodegenerative diseases (Khacho et al., 2019) (Figure 2). Neurons are highly dependent on mitochondria; 20% of the body’s oxygen is consumed by ATP produced by mitochondrial respiration in the brain (Attwell and Laughlin, 2001). Furthermore, the brain consumes 20% of the body’s total glucose intake, especially the nerve cells (Sokoloff, 1999). Although glycolysis can provide some ATP, most ATP is produced by mitochondrial respiration (Rangaraju et al., 2014). Mitochondria play an important role in the maintenance of physiological functions (Misgeld and Schwarz, 2017). Through continuous fission and fusion, a network structure with inner and outer membranes and multiple nucleoli containing mitochondrial DNA is formed (Braschi and McBride, 2010; Nabi et al., 2022). However, errors in mitochondrial fission or fusion can lead to nervous system diseases of varying degrees, and cognitive dysfunction is one of them. Pérez et al. (2018) reported that mitochondrial dysfunction is mediated by abnormal microtubule-associated proteins.

**FIGURE 3:**
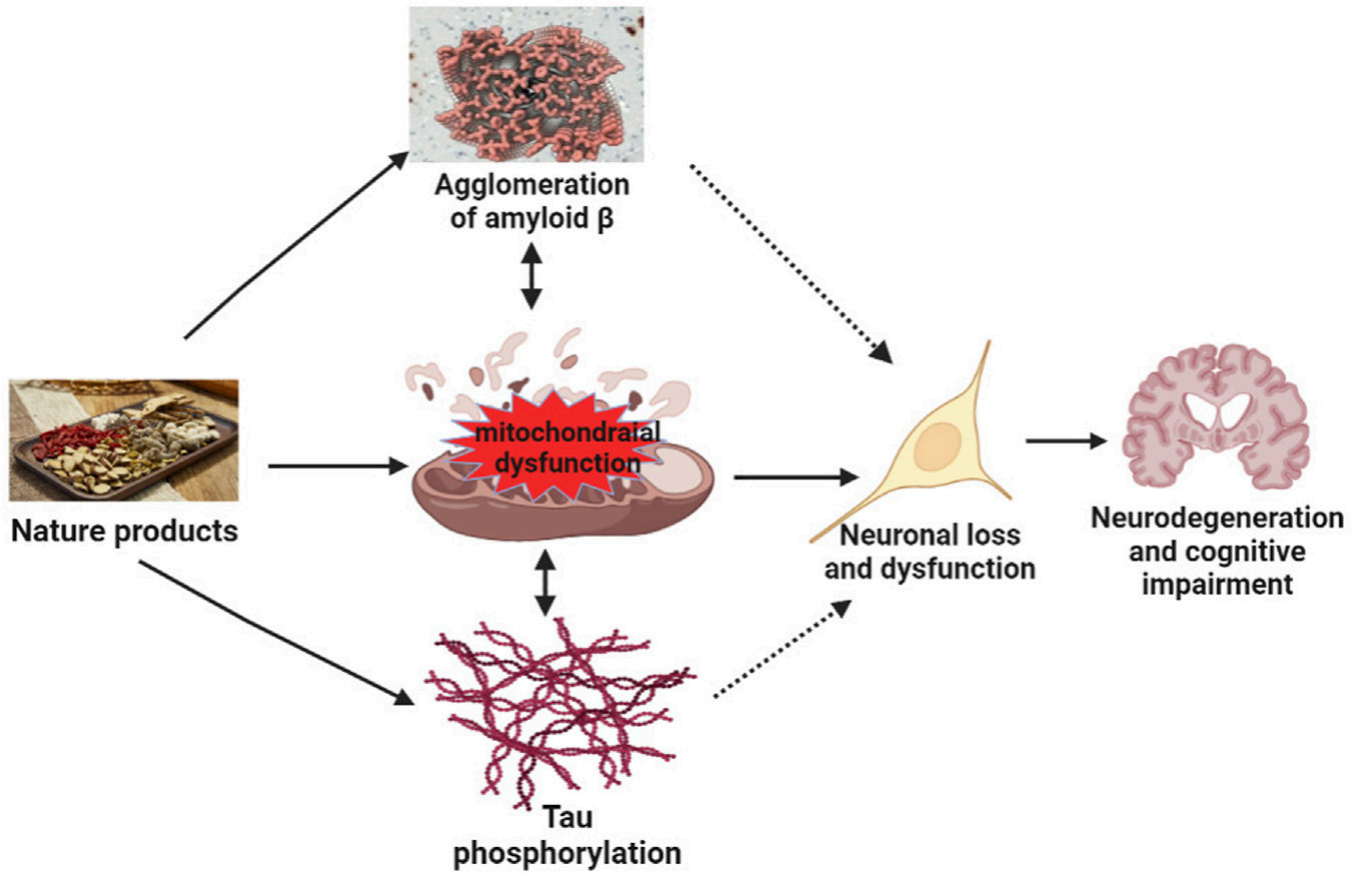
Mechanisms of natural products target mitochondrial function to improve neurodegeneration and cognitive impairment. Mitochondrial dysfunction results in the agglomeration of Aβ, tau phosphorylation, and agglomeration of Aβ and tau phosphorylation, further aggravating mitochondrial dysfunction, leading to neuronal loss or dysfunction, and ultimately inducing neurodegeneration and cognitive impairment. Natural products act on the mitochondria to improve tau phosphorylation and the agglomeration of amyloid β, neuronal loss, and dysfunction, thereby improving neurodegeneration and cognitive impairment.

At present, the pathogenesis of neurodegenerative diseases, such as AD, remains incomplete. Many recent studies have discussed the relationship between mitochondrial dysfunction and AD (Klein et al., 2021; Takeda et al., 2021). Tau is a key protein involved in the pathogenesis of AD. Pérez et al. (2018) showed that Tau pathology impaired mitochondrial transport affected mitochondrial dynamics and bioenergetics in AD. Multiple studies have shown that mitochondrial dysfunction is an early symptom of AD (Gibson and Shi, 2010; Cabezas-Opazo et al., 2015) and a driving factor of cognitive impairment in AD. Since mitochondria are easily affected by age, mutations, and metal toxins, dysfunction caused by mitochondrial DNA (mtDNA) damage, mutation, and impairment of metabolism and protein transport may lead to accumulation of Aβ oligomers or fibrils and phosphorylated Tau. Furthermore, the accumulation of damaged mtDNA and other macromolecules during aging leads to metabolic disorders (Abyadeh et al., 2021). The accumulation of damaged mitochondria further accelerates the progression of cognitive dysfunction (Swerdlow, 2018). The dysfunction of mitochondria further leads to the lack of bioenergy, the imbalance of intracellular calcium regulation, and the generation of free radicals. This causes oxidative stress and consequently to aggravation of Aβ oligomeric and Tau pathology, which, in turn, aggravates further synaptic dysfunction, mitochondrial damage, memory loss, and cognitive impairment (Figure 3).

Multiple studies have identified mitochondrial dysfunction as the central mechanism underlying many neurodegenerative diseases (Macdonald et al., 2018; Sharma et al., 2021). Significant progress has been made in understanding mitochondrial diseases, and there are now many treatments available for neurodegenerative diseases, including antioxidants, mitochondrial inducers, energy buffers, and gene therapy. However, satisfactory treatments are still lacking. Natural products have been used in various fields since the emergence of early antibiotics. Penicillin from mold, morphine from poppy, and paclitaxel, an anti-cancer drug, are typical examples of natural product drugs (Hunter, 2008). Natural product preparations may be an important breakthrough in the treatment of neurodegenerative diseases. Most natural products are extracted from microorganisms and exhibit a certain degree of cytotoxicity (Hunter, 2008). Therefore, when used clinically, they should be scientifically verified through clinical drug research at different levels.

The natural products discussed here mainly refer to the natural ingredients extracted from botanical drugs. An et al. (2021) reported that when DZXI (extracted from botanical drugs (Chai et al., 2013)) was administered to patients with acute ischemic stroke (AIS), the neurological and cognitive impairment of these patients was improved by regulating the mitochondrial respiratory chain and retaining the mitochondrial structure in brain tissue. Clinical studies have shown that DZXI treatment is beneficial for patients with AIS (Li et al., 2017). This study showed that the gray matter volume of the right anterior central gyrus, right central sulcus, right superior frontal gyrus, right middle frontal gyrus, and right inferior frontal gyrus in patients with AIS increased after DZXI treatment. Infarctions in these regions are associated with neurological and cognitive dysfunction (Galovic et al., 2013; Cheng et al., 2014; Wu et al., 2015; Henri-Bhargava et al., 2018; Parnaudeau et al., 2018; Zanto and Gazzaley, 2019; Zhu et al., 2019). Furthermore, DZXI treatment restored or reduced the volume of gray matter in specific areas such as the frontal cortex, which is a key area involved in cognitive dysfunction. In addition, DZXI treatment regulated the mitochondrial electron transport process, and the level of cytochrome C oxidase subunit 2 was significantly increased and protected the stability of neuronal mitochondria.

Among the studies retrieved in the present study, there are two articles about *Centella asiatica* (CAW), which is a plant that used in traditional Chinese medicine to enhance memory and improve cognitive function (Kapoor, 1990; Wattanathorn et al., 2008; Dev et al., 2009; Shinomol et al., 2011; Sirichoat et al., 2015; Gray et al., 2016; Umka Welbat et al., 2016; Chaisawang et al., 2017). In patients with epilepsy, long-term use of the current antiepileptic drugs cannot improve cognitive function (Ortinski and Meador, 2004; 77; Santulli et al., 2016). When CAW (*Centella asiatica*) is used to treat epileptic patients, it can not only alleviate seizures, but it also improves related memory disorders. The use of CAW before seizures has antiepileptic activity, which can restore the level of synaptophysin and mitochondrial function, reduce neuronal damage, and improve cognitive dysfunction. Therefore, natural medications are effective in treating cognitive dysfunction related to epilepsy. In conclusion, the natural product CAW may be beneficial for the treatment of epilepsy and related cognitive dysfunction.

In a mouse model of high-fat diet (HFD), the antioxidant activity of *Actinidia arguta* (CFAA) protected against HG-induced neurotoxicity and improved insulin resistance. At the same time, it was observed that the cognitive impairment was also improved through the reduction of oxidative stress and the enhancement of mitochondrial activity. CFAA can be used to treat neurodegenerative diseases caused by HFD.

Many studies on AD treatment have focused on natural products (Jeon et al., 2019). The present study collected five articles on the treatment of AD in patients with neurodegenerative dementia. Tetramethylpyrazine (TMP) from *Ligusticum chuanxiong* is a calcium antagonist with a strong neuroprotective effect in cerebral ischemia models (Chang et al., 2007). In rat models of Parkinson’s disease, TMP also has neuroprotective effects on dopaminergic neurons (Lu et al., 2014). In the AD mouse model of this study, after TMP treatment, mitochondrial proteins changed significantly, electron transport chain function improved, ATP levels increased, and synaptic dysfunction also improved.

The Ca^2+^ homeostasis in mitochondria is necessary to maintain normal neuronal function (Moreira et al., 2010). The protective effect of Ginkgolide K (GK) from *Ginkgo biloba* (a natural metabolite) on neuronal cells can promote neuronal cell survival (Liu et al., 2018), regulate mitochondrial function to exert a prosurvival effect (Ma et al., 2014; Zhou et al., 2017), inhibit MCU expression, reduce the Ca^2+^ level in the mitochondria, and prevent apoptosis.

Accumulation of Aβ in the brain is another important pathological feature of AD (Moon et al., 2012; Selkoe, 1994; Mancuso et al., 2009). Enhancement of mitochondrial homeostasis can offset Aβ protein toxicity and aggregation (Sorrentino et al., 2017), and *Red ginseng* (RGE) treatment can significantly restore impaired mitochondrial respiratory capacity and prevent mitochondrial fusion and division imbalance during Aβ-induced mitochondrial dysfunction. Simultaneously, RGE improved mitochondrial dysfunction and adult hippocampal neurogenesis, effectively preventing cognitive dysfunction in AD. The protective effect of RGE on mitochondrial function can reduce neuroinflammation. Therefore, RGE can promote AD therapy by protecting the neurons.

Rhein can improve mitochondrial function (Shin et al., 2019). Rhein activates SIRT1/PGC-1α, and mitochondrial biogenesis may be the key mechanism triggering the mitochondrial antioxidant defense system. After treating with Rhein, the expression level of SIRT1 and PGC-1α in the hippocampus of mice was significantly upregulated together with that of NRF1, which demonstrated that Rhein improved the biogenesis of mitochondria in AD mice, restored mitochondrial dynamics, repaired damaged mitochondria, and inhibited the electron transport chain to produce reactive oxygen species, thereby synergistically relieving neuronal oxidative stress (Yin et al., 2021). Thus, Rhein may have a potential therapeutic effect in AD.

The mixture of *Schisandra chinensis* extract (SCE) and ascorbic acid (AA) can improve cognitive function by enhancing mitochondrial respiration and inducing the activity of synaptic plasticity regulatory proteins, thereby improving mitochondrial function and memory and alleviating AD and age-related memory decline (Jang et al., 2020).

Sepsis, a common neurological complication, is associated with multiorgan failure, high mortality (Cecconi et al., 2018), and short-term or long-term neurological dysfunction (Silva et al., 2020). To date, there is no clear treatment. (−)-Epicatechin (Epi) from *Green tea* can be used as a new adjuvant treatment to improve the neurological prognosis of patients with sepsis. Epi is a natural polyphenol substance with an effective neuroprotective effect that has been shown to cross the blood-brain barrier and directly act on neurons (Mandel et al., 2008; Cruz-González et al., 2016; Bernatova, 2018). No obvious side effects have been observed (Borges et al., 2017). Epi controls the quality of mitochondria by activating AMPK signaling in the SAE. Epi treatment can reduce the decline of cognitive function, prevent the loss of neuronal dendritic spines, and reduce neuronal damage, which are related to the improvement of mitochondrial quality and reduction of neuroinflammation.

Cognitive dysfunction in the normal physiological process of aging is accompanied by various physiological phenomena (Beard et al., 2016; d'Avila et al., 2018). In the process of aging, there is dysfunction of organelles, such as mitochondrial enlargement or breakage; dysfunction of the electron transport chain; oxidative damage of mitochondrial DNA (Santos et al., 2013; Lores-Arnaiz et al., 2016). These processes may be important cell targets to protect against the cognitive dysfunction caused by aging. Trans-cinnamaldehyde (CIN) and ellagic acid (ELA) have been shown to inhibit oxidative stress, inflammation, apoptosis, and necrosis in many pathological models (Rajamani et al., 2015; Qi et al., 2016; Absalan et al., 2017; Firdaus et al., 2018; Rahimi et al., 2018). In this study, the combined treatment with ELA and CIN had a stronger and consistent effect on cognitive dysfunction in rats. Combined treatment can improve cognitive dysfunction induced by aging by inhibiting inflammation, protecting the mitochondrial function of the prefrontal cortex, and inhibiting the apoptosis signaling axis.

The main pathogenesis of vascular dementia (VD) includes chronic cerebral hypoperfusion (Damodaran et al., 2019), synaptic disorders (Sun, 2018), axonal abnormalities (Kalaria, 2018), hippocampal neuron loss (Jiang et al., 2019), and white matter vascular changes (Hase et al., 2019), which ultimately lead to cognitive impairment and dementia. The mitochondria are the main organelles involved in ischemia (Lv et al., 2017). Mitochondrial dysfunction in the brain plays an important role in the pathogenesis of VD. The SMYZD (which includes many Chinese botanical drug ingredients), can improve mitochondrial function, energy metabolism, and chronic cerebral perfusion insufficiency by activating the AMPK/PPARα/PGC-1α/UCP2 signaling pathway, which can improve mitochondrial structure and alleviate pathological damage in the rat brain. Anti-apoptotic and antioxidant properties, neurogenesis, and inhibition of mitochondrial dysfunction could exert these effects (Rajabian et al., 2019).

A previous study found that depletion of neuronal specific protein cell-cycle exit and neuronal differentiation 1 (CEND1) could lead to mitochondrial dysfunction, thereby causing cognitive dysfunction, and overexpression of CEND1 could improve cognitive dysfunction (Xie et al., 2022). It is unclear how natural products improve mitochondrial function under conditions of cognitive dysfunction, warranting further research to benefit the clinical treatment of cognitive dysfunction. Mounting evidence shows that the abnormality of mitochondrial dynamics generally precedes the hallmarks of pathology (Wang et al., 2021). Therefore, proper regulation of mitochondrial dynamics may be beneficial to AD patients.

The present study has limitations. The effect of natural products on mitochondria under conditions of cognitive dysfunction may not have been discussed comprehensively because we included few studies. Therefore, in the future, the inclusion criteria should be reconsidered and the mechanism of mitochondrial dysfunction in cognitive dysfunction should be discussed more comprehensively.

Overall, the mechanism underlying the effects of natural products on mitochondria is multileveled. The natural products used in clinical treatment are not only single botanical drugs but have multiple forms of composite applications. Whether there is drug interaction when they are used in combination remains to be determined. Therefore, metabolite botanical drugs, single botanical drugs, formulas, and combination of Chinese and Western medicine remain to be fully investigated to facilitate future clinical composite applications.

## 5 Conclusion

It is difficult for existing treatments to prevent the occurrence of cognitive dysfunction in neurodegenerative diseases; natural products may be a new therapeutic option. However, as natural botanical drugs are used for treatment, their mechanisms remain to be verified. The clinical use and in-depth research of natural products, especially the unique advantages in the field of cognitive impairment related to mitochondrial dysfunction, may create a new emphasis for drug research and the development of treatments for cognitive dysfunction-related diseases in the future.
