# Supplementary material for: Natural products regulate mitochondrial function in cognitive dysfunction—A scoping review
Source: Front Pharmacol. 2023 Mar 7;14:1091879. doi: 10.3389/fphar.2023.1091879 (PMC10027783; doi:10.3389/fphar.2023.1091879)
Supplement: Supplementary file 1 [file Table1.docx]

| **Supplemental file: Study characteristics** | | | | | |
| --- | --- | --- | --- | --- | --- |
| **Author/year** | **Country** | **Type of study** | **Name of**  **nature products** | **Mechanism of regulating mitochondrial function in cognitive dysfunction** | **Main results** |
| An et al.,  2019 | China | Clinical Trial  and  Animal Experiment | Dengzhanxixin Injection (DZXI) | The grey matter volume in specific regions in both human (stroke patients) and model animals was successfully restored by DZXI treatment.  DZXI treatment modulated mitochondrial electron transport chain process, significantly increased the levels of cytochrome c oxidase subunit 2 (COX2), and protected the mitochondrial stability in neural cells. | Dengzhanxixin injection treatment improved the cognitive functions of acute ischemic stroke patients the neuropsychological characteristics of the patients in each group were presented.  The protective effect of DZXI on mitochondria, which might allow the neural cells to become more resistant to ischemic injury. |
| Gray et al.,  2018 | USA | Animal Experiment | Centella asiatica (CAW) | Relatively short treatment with extract improved several different domains of cognitive performance in aged animals and enhanced synaptic density as well as mitochondrial and antioxidant response pathways in vivo. | Asiatic acid, a major triterpene component of Centella asiatica improves performance of cognitively impaired. |
| Lu et al.,  2021 | Taiwan | Animal Experiment | Centella asiatica | Asiatic acid might increase synaptic vesicle exocytosis by alleviating KA-induced presynaptic mitochondrial damage and increasing ATP production in hippocampal neurons, which may be crucial to maintain synaptic function and prevent cognitive deficits.  Asiatic acid may exert preventive effects against cognitive deficits in rats with KA-induced seizures through inhibiting glutamate release to increase AKT activation, inhibit calpain activation, and preserve synaptic and mitochondrial function. | Pretreatment with asiatic acid prevented such mitochondrial ultrastructural damage in the hippocampal synaptosomes of KA-treated rats with seizure.  12 proteins of hippocampal synaptosomes associated with the effect of asiatic acid, particularly its effects on the mitochondrial proteins related to energy generation. |
| Ha et al.,  2020 | Korea | Animal Experiment | Chloroform fraction actinidia arguta (CFAA) | HFD induced increased ROS production and decreased MMP and ATP in mitochondria, but the administration of CFAA improved HFD-induced mitochondrial dysfunction. | The cognitive dysfunction of HFD-induced insulin-resistant mice models was decreased, while in contrast, the CFAA groups improved compared with the HFD group.  Improved mitochondrial activity can also be evidence that CFAA is implicated in improving cognitive function. |
| Huang et al.,  2021 | China | Animal Experiment | Tetramethylpyrazine (TMP) | TMP creduces the accumulation of Aβ by inhibiting the activity of BACE1, which could affect levels of p-tau.  mitochondrial proteins were significantly changed after TMP treatment, with improved electron transport chain function and increased ATP levels in the brains of APP/PS1 mice, suggesting that mitochondrial function plays an important role in TMP treatment.  ARF6 may also be also involved in the neuroprotection of TMP. | TMP treatment significantly reduced the levels of Aβ accumulation and tau hyper phosphorylation in the two AD models relative to controls.  The increased ATP production after TMP treatment suggested that mitochondrial function was restored in APP/PS1 mice.  VAMP2 significantly increased after TMP treatment, suggesting that TMP treatment improves synaptic dysfunction. |
| Jang et al.,  2020 | Korea | Animal Experiment | Chinensis extract (SCE) and ascorbic acid (AA) | The SCE-AA mixture increased both PSD95 and GluR1—excitatory synaptic transmission regulatory proteins—in the hippocampus to improve cognition in mice through an enhancement of mitochondrial respiration.  The SCE-AA mixture can reduce the expression of PSD95 by short-term treatment. | The combination of SCE and AA enhanced mitochondrial respiration of hippocampal neurons and increased expression of key synaptic plasticity-related proteins in the hippocampus upon injection as a mixture (4:1 ratio) in mice.  GluR1 levels in the hippocampus were highest in mice treated with a mixture of SCE and AA, but they were also increased in mice injected with SCE or AA only. |
| Ling et al.,  2022 | China | Animal Experiment | (−)-Epicatechin  (epi) | The protective effect of Epi is mediated by the activation of AMPK signaling.  LPS interferes with mitochondrial function, leading to loss of mitochondrial membrane potential, energy stress, activation of inflammation, and cell death.  Epi may protect the mitochondria through the activation of AMPK signaling in SAE. | Epi upregulated pAMPK expression and that Epi lost its protective effect in AMPK-blocked cells.  Our study indicates a protective effect of Epi in LPS-induced cell and mouse models. |
| Liu et al.,  2022 | China | Animal Experiment | Ginkgolide K (GK) | GK treatment alleviated the increased expression of MCU induced by Aβ in vitro, which then decreased the Ca2+ levels in mitochondria and eventually inhibited the apoptosis of cells.The cognitive ability of APP/PS1 mice was clearly improved, with decreased expression of MCU in the neuronal cells of the mouse brain when GK was used to treat the AD mice.  GK exerted a prosurvival effect by regulating the function of mitochondria. | Increased Ca2+ levels in mitochondria resulted in a higher apoptosis rate of neuronal cells.  It was observed that treatment with Aβ resulted in apoptosis of neuronal cells, as well as increased Ca2+ levels in mitochondria.  GK promoted viability and prevented apoptosis of neuronal cells. |
| Ouyang et al.,  2022 | China | Animal Experiment | Capsaicin | This looser contact might be due to a decrease in MFN2 expression.  MFN2 at the molecular level supported this finding, although MFN2 ablation increased ER-mitochondria coupling.  Ischemia has a reducing effect on MFN2 expression.  The loosened MAMs and the low expression levels of MFN2 detected in CCH rats might suggest a mechanism that induces cognitive impairment.  Capsaicin would exert a neuroprotective effect on cognitive deficits induced by CCH. | CCH rats have early and long lasting cognitive impairments, as manifested by decreases in both spatial learning and memory, as well as short-term recognition and non-associative learning impairments accompanied by anxiety-like emotion, in studies using the MWM, ORT, and OFT.  A statistically significant decrease in the distance of ER and mitochondrial contacts in only the 4-week groups using electron microscopy.  A decrease in the proportion of ER-mitochondria associations relative to the total mitochondrial circumference in CCH compared to the sham group at each time point, indicating that ER and mitochondrial tethering in CCH exhibited a looser state. |
| Pan et al.,  2020 | China | Animal Experiment | Trans-cinnamaldehyde (CIN) and ellagic acid (ELA) | Combination therapy could significantly improve mitochondrial function by reducing the mitochondrial ROS production and mitochondrial membrane depolarization, increasing cellular ATP production, declining inflammatory cytokines (IL-1β and IL-6), and lessening cell apoptosis by reducing Bax/Bcl2 ratio and cleaved-caspase 3 expression in the prefrontal cortex of aged rats. | The CIN alone improved the cognitive function of the old rats compared to the ELA and control groups. In addition, the combination of these two drugs could have stronger and consistent effects on the parameters related to the cognitive function of rats. |
| Shin et al.,  2019 | Korea | Animal Experiment | Red ginseng extract (RGE) | RGE ameliorates mitochondrial dysfunction and Aβ-mediated pathologies, including Aβ deposition, gliosis, and neuronal loss, and decreased adult hippocampal neurogenesis in 5XFAD mice, an animal model of AD.  RGE does not simply enhance the mitochondrial respiratory function, but also prevents Aβ deposition and Aβ-related pathologies directly or indirectly by mediating the recovery of mitochondrial functions.  RGE ameliorated the mitochondrial dysfunction as well as the adult hippocampal neurogenesis. | Treatment with RGE significantly restored the impaired mitochondrial respiratory capacity and protected against the imbalance in mitochondrial fusion and fission during Aβ-induced mitochondrial dysfunction.  A significant reduction in the 4G8 (+) area was observed in 5XFAD mice treated with RGE.  5XFAD mice that received RGE showed a significantly increased number of Ki-67 (+) cells than did the WT mice. |
| Sun et al.,  2021 | China | Animal Experiment | ShenmaYizhi decoction (SMYZD) | The therapeutic effect of SMYZD is achieved by activating the AMPK/PPARα/PGC-1α/UCP2 signalling pathway to improve mitochondrial structure and alleviate pathological injury and oxidative stress-induced injury in the brains of VCI rats. This treatment may restore mitochondrial structure and improve mitochondrial function by inhibiting oxidative stress and exerts a synergistic effect on improving the cognition of VCI rats. | SMYZD improves mitochondrial structure and energy metabolism to ameliorate chronic cerebral hypoperfusion.  Decreased expression of pAMPK, PPARα, PGC-1α, and UCP2 in the mitochondria of the brains of VCI rats was detected, accompanied by pathological changes in the mitochondria. |
| Yin et al.,  2022 | China | Animal Experiment | Rhein | Mitochondrial fission proteins like DRP1 contribute to regulating the number of mitochondria, and fusion proteins like MFN1 help to assure the quality of mitochondria.  Due to the improved mitochondrial biogenesis by rhein, the recovery of mitochondrial dynamics to repair damaged mitochondria and inhibit the production of ROS from ETC. | The rhein improved mitochondrial biogenesis, newly generated healthy mitochondria expressed the decreased expression of DRP1 and increased expression of MFN1 to a normal level. |
| Yin et al.,  2021 | China | Animal Experiment | Rhein, emodin, aloe-emodin, chrysophanol, and physcion | Rhein reduced the release of cyto c from mitochondria, inhibiting the apoptosis cascade and ultimately protecting neurons from apoptosis.  Rhein played a positive role in regulating both the enzymes in mitochondrial respiratory chain complexes and antioxidant enzymes, suggesting that it may be beneficial to improve mitochondrial biogenesis.  Rhein activates mitochondrial biogenesis regulated by the SIRT1/PGC-1α pathway as an antioxidant defense system against Aβ1-42 oligomer-induced oxidative stress. | Among all the five anthraquinones, rhein possessed excellent antioxidant activity.  The levels of cytosolic cyto c and cleaved caspase 3 were reversed after rhein treatment.  The activity of CytOx increased after treatment with rhein, and SOD activity was significantly increased under the treatment of rhein. |
| KA: kainic acid; AKT: protein kinase B; HFD: high-fat diet; ROS: reactive oxygen species; MMP: mitochondrial membrane potential; ATP: adenosine triphosphate; BACE: Beta-secretase; ARF: ADP ribosylation factor; PSD: postsynaptic density protein; MCU: mitochondrial Ca2+ uniporter; AD: Alzheimer's disease; MAMs: Mitochondria-associated endoplasmic reticulum membranes; MFN: expression of mitofusin; ER: endoplasmic reticulum; CCH: chronic cerebral hypoperfusion; VCI: vascular cognitive impairment; DRP: dynamin-related protein; ETC: electron transport chain; CytOx: cytochrome c oxidase; SOD: superoxide dismutase. | | | | | |
|  | | | | | |
